# Factors influencing novice supervisors’ trust decisions in postgraduate family medicine training: a qualitative study

**DOI:** 10.3389/fmed.2026.1712735

**Published:** 2026-02-04

**Authors:** Aminah Al-Sulaiman, Sylvia Heeneman, Muhammad Zafar Iqbal, Cees van der Vleuten

**Affiliations:** 1 Family Medicine Academy, Al Khobar, Saudi Arabia; 2 Department of Pathology, School of Health Professions Education, Cardiovascular Research Institute Maastricht, Maastricht University, Maastricht, Netherlands; 3 Department of Research, Acuity Insights Inc., Toronto, ON, Canada; 4 Department of Medical Education, University College of Medicine & Dentistry, The University of Lahore, Lahore, Pakistan; 5 School of Health Professions Education, Maastricht University, Maastricht, Netherlands

**Keywords:** clinical training, family medicine, novice supervisors, postgraduate trainees, trust building

## Abstract

**Background:**

Trust is a foundational element in clinical supervision, influencing how and when autonomy is granted to postgraduate trainees. While prior research has largely focused on experienced supervisors, less is known about how novice supervisors navigate trust decisions.

**Objective:**

This study aimed to explore how novice supervisors in postgraduate family medicine perceive trust and what factors influence their trust-building process.

**Methods:**

In 2022–23, a qualitative descriptive study was conducted to examine trust formation among novice supervisors in a postgraduate family medicine training program in Saudi Arabia. Twenty supervisors with less than 5 years of supervisory experience were interviewed using semi-structured, one-on-one interviews. Data were analyzed using a framework analysis approach, guided by the model of trust formation.

**Results:**

Supervisors described trust as an evolving process shaped by cumulative observations, perceived clinical risk, and accountability. Initial interactions were characterized by high supervision and low trust, which adjusted over time through repeated exposure and relationship building. Institutional challenges such as short rotations, legal constraints, and high workloads often disrupted trust development, sometimes leading to superficial or “fake” entrustment. Supervisors with more clinical experience were more confident in calibrating trust, whereas less experienced supervisors often relied on cautious, checklist-style judgments.

**Conclusion:**

Novice supervisors build trust incrementally through experience, observation, and relational engagement, but are often constrained by systemic and contextual barriers. Faculty development programs, structural supports and longer rotation periods may enhance trust calibration and enable safer, developmentally appropriate supervision.

## Introduction

A key objective of postgraduate clinical training programs is to prepare trainees for competent and unsupervised practice. The transition from direct supervision to unsupervised clinical practice hinges critically on a supervisor’s willingness to trust trainees with autonomous patient care (1). In postgraduate family medicine training where trainees are expected to handle complex and uncertain situations, trust becomes a vital construct that modulates both supervision and learning. Without trust, trainees may remain passive observers, whereas prematurely granted trust may endanger patient safety (2). Therefore, supervision in postgraduate training involves a delicate balance between ensuring safe, high-quality patient care and progressively granting trainees greater autonomy. At the heart of this balance lies trust, a dynamic, multifaceted judgment that supervisors must make daily about their trainees’ readiness to perform independently (1–4).

Trust, though commonly understood, lacks a universally agreed-upon definition due to its context-dependent nature. Fundamentally, trust arises from the need for social cooperation and is evident in diverse relationships, including those among humans, animals, and within systems requiring interdependence. Trust allows individuals to navigate complexity, collaborate effectively, and develop within social structures (5, 6). Whether acquired through experience or innate tendencies, trust plays a crucial role in personal development, enabling individuals to engage effectively in relationships, communities, and broader societal systems (7). In clinical education, trust is defined as the belief that someone is reliable, competent, and has good intentions (8), and it plays a critical role in balancing patient safety with trainee learning.

The development of trust is shaped by multiple interrelated factors, including the supervisor’s clinical and educational expertise, the trainee’s performance and behavior, the nature of the task, the clinical context, and the quality of the supervisor–trainee relationship (1, 9, 10). These five domains form the conceptual core of most empirical and theoretical models of entrustment in medical education (1, 2, 6). For example, supervisors’ own confidence in their clinical abilities and teaching skills influences how readily they relinquish control (4). Their attitudes, experiences, and even dispositional propensity to trust contribute to entrustment decisions (11).

Recent medical education literature has moved beyond clinical competence alone to define trust as a multidimensional construct involving both cognitive appraisal and affective dimensions (1, 10). Supervisors consider not only knowledge and technical skills of the trainees but also their personality traits like integrity, self-awareness, professionalism and help-seeking behavior (12–14). Trust often begins with thin-slice impressions and is refined through ongoing interaction and performance observation. Studies suggest that supervisors’ initial trust judgments are shaped by brief early interactions and refined over time through continued observation, comparison with normative standards, and sometimes feedback from other team members or patients (15–17). When appropriately calibrated, trust enables learning through participation, helping trainees move from peripheral involvement to legitimate roles within a community of practice (1, 18).

The supervisor–trainee relationship itself acts as a conduit for trust development. Relational signaling theory suggests that trust is co-constructed through shared expectations, interpersonal rapport and mutual respect, and behavioral cues such as openness and feedback responsiveness further facilitate the trust-building process (19, 20). In short-term rotations, lack of familiarity may push supervisors to rely on heuristics, while long-term supervision enables more grounded, longitudinal assessment and trustworthiness of the trainee (9, 21). Furthermore, ambiguity in supervisors’ dual roles as mentors and assessors can distort trust judgments, sometimes leading to inflated ratings or avoidance of critical feedback (9).

Task-specific and contextual (i.e., training environment and institutional culture) variables further modulate trust. Task complexity and risk level are particularly important in entrustment decisions. Simple tasks with low variability and minimal consequence may warrant early trust, whereas complex or high-stakes situations demand more experience and supervision (4, 11, 22). In this context, the “zone of proximal development” provides a developmental framework, suggesting that supervisors should scaffold autonomy within appropriate bounds (2, 23). With respect to the context, high workload, patient acuity, and lack of systemic support may either hamper the trust-building process or prompt premature delegation under pressure (1, 24). Conversely, structured supervision opportunities for graded responsibility aim to support safe and developmentally appropriate entrustment (18, 25).

Novice supervisors, in particular, face unique challenges in making trust-related decisions, as they often lack the experience and confidence that seasoned supervisors possess. In comparison to seasoned clinicians, novice supervisors often tend to rely on rule-based supervision, focusing on checklist-style assessments and task completion as they might experience uncertainty in assessing competence, or struggle to balance supervisory vigilance with fostering autonomy (4, 9, 10). According to Dreyfus’ model of skill acquisition (26), expertise evolves from rigid rule-following to intuitive, context-rich interpretation. With experience, supervisors gain a more nuanced understanding of context, learner trajectory, and relational cues (22). Although recent studies have advanced understanding of supervisor trust, most research focuses on seasoned clinicians. Little is known about how novice supervisors, especially in primary care settings like family medicine, conceptualize and enact trust. This knowledge gap is significant because trust underpins a trainee’s access to clinical experiences necessary for competence development and independent practice. Therefore, this study seeks to explore how novice supervisors in family medicine perceive trust and what factors influence their trust-building process.

## Materials and methods

### Study design

This study employed a qualitative descriptive design (27) to explore how novice supervisors in postgraduate family medicine perceive trust and what factors influence their trust-building process. A qualitative descriptive approach is well-suited for capturing participants’ views and experiences in their own words (27). It offers a flexible yet structured approach to exploring real-world phenomena, particularly in applied settings such as the one examined in this study.

### Training context and study setting

This study was conducted at the Family Medicine Academy (FMA), a governmental teaching institution under the Ministry of Health (MOH) in the Kingdom of Saudi Arabia (KSA). The FMA is a major provider of the Family Medicine Postgraduate Program (FMPGP), a 3-years, competency-based residency program governed by the Saudi Commission for Health Specialties (SCFHS) and implemented across multiple institutions nationwide.

The FMPGP is structured around rotations in two primary settings: hospital-based specialty rotations and Ambulatory Family Medicine Clinics (AFMCs) located in Primary Healthcare Centers (PHCCs). Supervisors affiliated with the FMA are involved with trainees exclusively in the AFMC setting, which spans 30 PHCCs distributed across three cities in the Eastern Province. Supervisor–trainee contact in this setting is limited to discrete 3-months rotations, after which trainees rotate to another PHCC or to a hospital-based placement. As a result, supervisory relationships are typically short-term and discontinuous, limiting opportunities for longitudinal trainee-supervisor interactions.

Assessment within the FMPGP relies on multiple workplace-based assessment tools, including the Mini-Clinical Evaluation Exercise (Mini-CEX), Direct Observation of Procedural Skills (DOPS), and Case-Based Discussion (CBD). Entrustable Professional Activities (EPAs) are not formally incorporated into the program’s assessment framework. In addition, trainees’ electronic medical record privileges are restricted and require supervisory oversight.

Although the FMPGP is implemented across several institutions, this study was conducted exclusively at the FMA. The FMA hosts the largest number of family medicine supervisors nationally and the highest proportion of novice supervisors. This context provided a particularly relevant setting for exploring how novice supervisors develop trust within structurally constrained, time-limited supervisory relationships.

All trainees entering the program hold a medical degree; however, their prior clinical experience varies, ranging from recent medical school graduates to physicians with previous hospital or primary care experience. This variability further contributes to the complexity of supervisory trust judgments within the program.

### Study participants

The Family Medicine Academy (FMA) has the highest number of supervisors (*N* = 65) in the Kingdom, as well as the highest proportion of novice supervisors (*N* = 41) – defined as those with less than 5 years of supervisory experience – compared to other institutions. Among these 41 novice supervisors, 15 were male and 26 were female. These novice supervisors had joined the FMA immediately after completing their residency or had only brief clinical experience, and none had received formal training or held a background in medical education. All eligible supervisors were invited via email to participate in the study, which included a written informed consent form outlining the study background, objectives, expectations, potential risks, and a confidentiality statement.

### Data collection

Principal investigator (AA) collected the data between September 2022 and January 2023, using semi-structured, one-on-one interviews with novice supervisors. Each interview lasted a maximum of 40 min. Participants were informed that they could speak freely, that the session would be audio-recorded, and that all information would remain confidential. A set of open-ended questions, developed based on the model of trust formation (2), served as the interview guide (see Supplementary Appendix 1). The interview guide was piloted with two supervisors not included in the final sample. Minor wording adjustments were made to improve clarity of the questions. Interview guide was also reviewed and approved by the co-authors before formal data collection. All interviews were transcribed verbatim and member checking was conducted, with participants providing minor comments and modifications to the transcripts (28). Member checking involved returning transcripts and preliminary interpretations to participants, which was particularly important because participants frequently switched between Arabic and English during interviews, and this process helped ensure that meanings, nuances, and contextual subtleties were accurately captured during translation. Some supervisors provided clarifications about supervisory constraints and contextual nuances, which led to refinement of analytic categories and strengthened interpretive accuracy.

### Data analysis

Data were analyzed using a framework analysis approach (29), supported by ATLAS.ti (version 22) to facilitate the systematic organization and coding of the data. Framework analysis was selected because it allows for the structured examination of qualitative data while remaining transparent, systematic, and theoretically informed. The analysis was guided by the model of trust formation proposed by Huaer et al. (2), which identifies five overarching domains influencing trust: supervisor characteristics, trainee characteristics, supervisor–trainee relationship, context, and task complexity.

The principal investigator (AA) conducted initial coding of all interview transcripts using these five domains as a predefined analytical framework. This deductive coding approach involved indexing segments of text to the relevant framework domains while remaining attentive to variations in how participants described trust formation within each domain. The research team members (SH and CvdM) then collaboratively reviewed and refined the coding through synchronous discussions (via Zoom) and asynchronous correspondence (via email), ensuring consistency and enhancing the credibility of the analysis. Discrepancies in coding were discussed, resulting in two rounds of code revision conducted by the principal investigator (AA) to ensure accurate representation of the data.

Following the principles of framework analysis (29), data were organized using a matrix-based approach, allowing for systematic comparison of participants’ responses across the five domains. This process involved charting summarized data into a framework matrix, with cases represented in rows and framework domains represented in columns. This supported the identification of patterns, similarities, and differences both within and across cases. The matrix facilitated higher-level interpretation by enabling the research team to explore relationships among domains and to generate analytically grounded insights into trust formation.

Analysis of the 20 interviews provided sufficient depth and redundancy to support the identified themes within the predefined framework domains. Rather than claiming full data saturation, the study achieved thematic sufficiency within the participating sample, acknowledging that views of non-participants may not be fully represented.

## Results

Of the 41 invited supervisors, 20 (6 males and 14 females) agreed to participate and were interviewed. Participants’ clinical experience post-graduation ranged from 2 to 13 years, with a mean of 4.45 years. Their ages ranged from 31 to 46 years, with a mean of 37.5 years. The mean duration of their experience as family medicine supervisors ranged from 1 to 4.5 years, with a mean of 2.55 years.

All key findings were found relevant to the five themes highlighted in the model of trust formation (theoretical framework), reflecting the complex and context-dependent nature of trust decisions among novice supervisors. In addition, a cross-cutting theme, “Meaning of Trust,” consistently emerged, illustrating how trust is constructed as a fluid, evolving process shaped by individual, interpersonal, and organizational factors. Although the analysis was guided by the five-domain trust framework, the theme “Meaning of Trust” reflects a higher-order construct that captures participants’ underlying conceptualizations of trust, which in turn shaped how they enacted trust judgments across all five domains. Table 1 summarizes key themes and associated factors.

**TABLE 1:** Themes and sub-themes of factors Influencing trust decisions among novice supervisors.

Themes	Sub-themes or factors
Meaning of Trust	- Evolving and dynamic process – Awareness of trainee competence through repeated observation
Supervisor characteristics	- More supervision/less trust approach at start of training – Legal accountability and fear of complaints – Experience level influencing trust propensity – Struggles with supervisory style (gatekeeper vs. guide)
Trainee characteristics	- Clinical reasoning and decision-making quality – Professionalism, punctuality, commitment – Year of training and prior familiarity – “Red flags” (e.g., poor judgment, overconfidence, low-level questions) – Self-reflection, positive attitude
Supervisor–trainee relationship	- Rapport and relationship-building – Extent and duration of contact – Impact of frequent rotations – Expectation alignment
Contextual factors	- Workload and staffing shortages – High trainee-to-supervisor ratio – Legal constraints and limited trainee privileges – Disorganized clinical environment – Cultural norms and institutional barriers
Task complexity	- Integration of trainee level, competence, and task risk – More trust in routine, recurrent tasks – Reluctance in high-risk or complex tasks – Use of task sequencing to scaffold autonomy

### Meaning of Trust

Participants described trust as an awareness of a trainee’s competence based on repeated observation, leading to the conclusion that the trainee was a safe doctor. Trust was commonly framed in terms of patient safety, clinical judgment, and the ability to recognize and manage urgent or complex situations.

*“Trust has many parameters. The doctor must be a safe doctor through ruling out the emergency, urgency, and seriousness of the condition. The doctor must have a good knowledge scientifically, medically and clinically so that he can perform competently.”* (T10)

Across interviews, trust was not perceived as a one-time or binary decision, but rather as a dynamic and evolving process, particularly during the early stages of the supervisory relationship. Supervisors described an initial period of uncertainty, often referred to as a “gray zone,” during which trust judgments remained provisional and continued to shift based on ongoing performance, observation, and clinical judgment.

*“Well, it [trust] is not an abrupt decision that has to be made on a hunch. Rather, it is a continuous back and forth decision, and it is mostly a gray zone until I feel that the resident is capable and ready.”* (T13)

Participants’ descriptions of trust reflected underlying assumptions about responsibility, risk, and readiness for independent practice, which informed how novice supervisors approach entrustment decisions across different situations and supervisory contexts.

### Supervisor characteristics

In the early stages of training, supervisors described being cautious with new trainees, adopting the “more supervision/less trust” approach regardless of the trainee’s academic credentials. The sense of accountability for the patients’ and trainee’s actions led to more supervision and complaint-avoidance behaviors among supervisors, reflecting a legal constraint. One participant mentioned:

*“At the end of the day, if something goes wrong, it is my name and my career at stake. So, I am careful about how much I can trust them.”* (T19)

Some supervisors mentioned they would not leave even senior residents unsupervised until they observed specific attitudes or behaviors that warranted trust. This reflects less propensity to trust:

*“There are certain criteria I have to see before giving full responsibility, but usually, they have to consult me. For any new trainees, I keep them attached. Never alone even if they are senior, until I notice a specific level of progress in performance…” (T7)*

Supervisors with more clinical experience described a greater tendency to trust, framing their role more as a guide than as a gatekeeper. They were more likely to include trainees in decision-making and encourage participation while maintaining oversight. Many described trust as linked to their perceived responsibility for the patient’s outcome, which led to behaviors aimed at avoiding complaints or clinical errors.

*“Well, I do trust the majority of residents to be a large part or contributor to the responsibilities of patient care. However, they will always be guided in my presence…”* (T13)

### Trainee characteristics

Supervisors identified trainee characteristics as central to their trust decisions. Direct observation played a significant role, whether through clinical consultations, medical documentation, or case discussions. Supervisors assessed clinical knowledge, reasoning, and the complexity of the questions trainees asked. Additional input from co-workers, patients, and other supervisors also contributed to trust formation. Specific behaviors were closely monitored, including punctuality, commitment, and professionalism. Supervisors noted that a trainee’s level of training influenced their trust, with senior trainees generally being trusted more. Familiarity with a trainee (i.e., having previously worked together) reduced uncertainty and helped build trust more quickly.

Supervisors also described “red flags” that triggered hesitation or loss of trust. These included poor clinical reasoning, asking questions below the expected level, over-confidence or failing to recognize the seriousness of a case.

*“One time, a year 4 resident that I expected to perform good, asked a question that was simple in the [patient] management, so I was surprised that he has no knowledge to make the decision. The question was a year 2 level. So, here I hesitated to trust him.”* (T9)

On the other hand, qualities like self-awareness, self-confidence, positive attitude, prior work experience, and the ability to self-reflect were also reported as influential factors.

### Supervisor-trainee relationship

Factors affecting trust in supervisor-trainee relationships included relationship formation, extent of contact and expectation alignment. Relationship formation through rapport-building and the creation of a safe learning environment, was described as an important influence on trust decisions. The extent of contact was also considered highly crucial for trust building, as longer durations of interaction were seen to foster greater trust. However, the structure of the program limited each supervisor-trainee relationship to 3 months out of the trainee’s 3-years training, which impacted both learning and trust development. This short duration often disrupted the continuity needed to establish trust. Frequent rotations across centers meant that trainees were repeatedly viewed as “new” by different supervisors, requiring additional time to build trust each time.

Expectation alignment was discussed more frequently by clinically experienced supervisors, who emphasized the need to adapt supervision styles to match individual trainee expectations. As one supervisor explained:

*“The resident comes with an expectation that might be different from us [supervisors]. So, your supervision style should be like a key that opens the specific combination of your resident’s skillset.”* (T18)

In contrast, less clinically experienced supervisors described expectations by focusing more on setting clear boundaries around clinical tasks to support independent practice and avoiding complaints.

### Context

Participants identified workload, legal constraints and organizational barriers as crucial factors influencing their trust decisions. Less commonly mentioned factors included workplace affordances, familiarity with the healthcare system, staff competency, and workplace culture. Staff shortages, particularly among physicians, and a high trainee-to-supervisor ratio were key contextual features that influenced trust. These shortages increased the workload for trainees, often leading to chaotic and disorganized workplace conditions. One participant mentioned:

*“The crowded, disorganized situations. I get worried that I can’t trust them in the crowd. They might have judgment issues.”* (T8)

Several supervisors described deliberately extending autonomy to trainees despite lingering uncertainty about their readiness, a practice they explicitly referred to as “fake trust” and discussed as a pragmatic response to deal with the high patient influx, which they found unsatisfying. One participant mentioned:

*“Sometimes I have no choice but to let them work on their own because there are too many patients to handle. I don’t feel comfortable with this, but what other option I have.”* (T10)

Several legal constraints also impacted trust building. One common example was the restriction on tasks trainees could perform (e.g., writing patient reports, referring patients, or prescribing medications) due to limited electronic account privileges. Moreover, since supervisors are legally responsible for trainees’ actions, they reported reduced trust and increased supervision, describing the need for a “leap of faith.”

### Task complexity

Task complexity and the level of patient complexity (including the severity of medical, psychosocial, or communication issues) affected the trust of novice supervisors. Participants reported that they often based trust decisions on the integration of three key elements: perceived trainee competence, the trainee’s level or year of training, and the complexity of the patient or task. The findings also indicated that supervisors were more likely to trust trainees with routine and recurrent tasks than with unfamiliar or new ones.

*“The more recurrent a task, the easier to trust; for example, a newborn baby examination. It is a bit complex with a lot of checks and steps but because of the recurrence, I trust them because we might see up to 7 cases per week.”* (T17)

Sequencing tasks to provide trainees with appropriately challenging tasks for their level was also described. As one participant said:

*“Sometimes, I challenge my students with a bit complex task. Once, I had a resident in OB/GYN. She attended with me three cases for ultrasound. I did it once myself and for the second patient, I ask her to do the ultrasound, and she did it perfectly. So, the third time an antenatal case came up, I trusted her, and I was sure that she will perform well.”* (T6)

## Discussion

This qualitative study explores the strategies that novice supervisors employ and the multifactorial influences on their trust decisions regarding trainees’ independent clinical practice. Findings illustrate that novice supervisors perceive trust not as a static threshold but as a dynamic, evolving process shaped by cumulative experiences, contextual contingencies, and relational factors (1, 2).

While the five-domain model provided a robust structure for examining factors influencing trust decisions, our findings suggest that novice supervisors’ conceptualization of what “trust” means operates as an overarching interpretive lens rather than a discrete domain. For many participants, trust was understood less as a binary decision and more as a pragmatic, risk-management strategy shaped by experience, accountability, and context. This suggests that existing models may benefit from greater attention to how supervisors’ underlying trust definitions shape the application of domain-specific judgments, particularly among novice supervisors.

At the initiation of a supervisory relationship, novice supervisors commonly adopted a “more supervision/less trust” approach, often rooted in limited familiarity with the trainee and concern for patient safety (1, 30). This behavior reflects early developmental stages of supervisor expertise, where decisions are rule-based and detail-oriented rather than holistic (9). Notably, the transition from observation to entrustment was contingent on repeated, meaningful interactions that fostered familiarity and confidence in the trainee’s reliability (6, 10, 19).

Trust facilitators and barriers were identified across several dimensions. Novice supervisors’ own propensity to trust, shaped by clinical experience, personal disposition, and institutional culture, played a critical role. As noted by Hancock et al., dispositional trust is a foundational individual trait that affects how supervisors interpret ambiguous trainee behaviors (11). Novice supervisors with greater clinical experience exhibited higher baseline trust, potentially due to enhanced metacognitive skills and confidence in risk calibration (12).

Trainee-specific factors (including clinical competence, communication skills, insight, and receptivity to feedback) were consistently noted as significant to trust development. Importantly, novice supervisors often placed emphasis on behavioral predictability and task completion over nuanced clinical reasoning, reflecting limited trust assessment literacy. This reliance on discrete observations is typical of early-stage supervisors, who may lack the experience to synthesize contextual and behavioral cues into an integrated trust assessment (6, 10).

The supervisor–trainee relationship emerged as central to trust calibration. When a sense of psychological safety and open communication was established, novice supervisors were more likely to afford autonomy. This corresponds with the concept of mutual trust as a reciprocal, socially situated construct that underpins collaborative learning (1, 18). However, many novice supervisors struggled with the dual role of assessor and coach, a tension that risked relational strain or role confusion. This is a common challenge identified in the literature as relationship interference in assessment contexts (31, 32).

Contextual barriers such as high patient loads, inadequate staffing, and unpredictable workflow, particularly in primary healthcare centers, significantly constrained novice supervisors’ ability to observe and mentor trainees effectively. This often led to pragmatic compromises such as “fake trust,” where supervisors allowed trainees to operate semi-independently without full confidence in their readiness, prioritizing throughput over educational fidelity. Some novice supervisors opted to minimize risk by retaining control of complex tasks and limiting trainee responsibility, particularly in high-stakes or unfamiliar clinical scenarios. While this approach may preserve patient safety, it may also constrain opportunities for trainee development (33, 34). These findings align with previous work describing trust as relational and risk-calibrated (1, 2, 19). The practice of “fake trust,” in which autonomy is granted for workflow expediency rather than developmental readiness, echoes concerns raised in studies of entrustment under system pressures (33). Accountability-related anxieties documented in our study also resonate with work identifying legal and reputational risks as significant moderators of trust decisions (4, 11). Our findings therefore extend current theory by demonstrating how these tensions manifest uniquely among novice supervisors in short-rotation primary care contexts.

Task complexity, particularly in high-risk or unfamiliar scenarios, elicited the most conservative trust behaviors. Regardless of trainee seniority, novice supervisors exercised significant caution with complex or unpredictable patients, echoing well-documented trust asymmetries in risk-laden contexts (2, 10, 12). Supervisors were more willing to entrust routine and recurrent tasks, especially those they had previously observed the trainee perform. This reinforces the importance of structured observation and feedback mechanisms in cultivating progressive autonomy (19).

Interestingly, while some novice supervisors with more experience discussed aligning expectations explicitly with trainees (a practice known to support goal clarity and feedback utility), others failed to do so. The absence of expectation-setting mechanisms undermines tailored feedback and can erode mutual understanding between supervisors and their trainees.

### Implications and recommendations

The findings of this study have important implications for faculty development, curriculum design, and institutional policy. Novice supervisors may benefit from structured training that integrates observation strategies, feedback delivery, and entrustment decision-making frameworks (e.g., EPA-based models). Enhancing supervisors’ capacity through training to assess performance and align expectations with trainees could lead to more accurate and developmentally appropriate trust decisions. Faculty development programs should explicitly address how to assess readiness for autonomy and how to balance patient safety with graduated responsibility, using EPA-based tools and scenarios. Based on identified barriers, novice supervisors might further benefit from partnering with more experienced supervisors, particularly in their initial months of supervisory responsibility, to support decision-making in ambiguous or high-stakes scenarios.

Furthermore, prolonging supervisor–trainee relationships by extending clinical rotation durations to 6 months or more would allow trust to be built on deeper, more contextualized observations, thus supporting more grounded and defensible trust decisions. Moreover, implementing systems where trainees are allowed to manage a limited number of patients independently under indirect supervision may help novice supervisors avoid the phenomenon of “fake trust” in which trainees are given autonomy prematurely due to workload constraints. This approach may also help reduce role compression dynamics, in which supervisors or trainees take on responsibilities misaligned with their intended scope of practice (e.g., residents being treated as interns or attendings assuming routine resident tasks).

At the systems level, addressing contextual stressors such as high supervisor–trainee ratios and inconsistent patient care continuity can enable more authentic supervisory relationships and reduce the likelihood of compromised trust trajectories. Supporting supervisors to align their supervision styles to developmental goals, rather than institutional pressures, can improve learning, safety, and professional identity formation.

### Limitations and future research

This study has several limitations. First, all participants were recruited from a single governmental training institution. Moreover, we interviewed approximately 49% of the novice supervisors available in the selected institution, which may have helped us capture sufficient nuances in the trust-building process. However, the perspectives of those who were unable to participate in the study may differ from those reported here; therefore, the findings have limited generalizability. As institutional cultures, supervisory structures, and clinical workloads differ widely, future research could include a broader and more diverse sample of novice supervisors from multiple centers (including private and academic medical centers) and disciplines (i.e., surgery, pediatrics, internal medicine etc.) to examine whether the factors influencing trust-building process identified in this study are consistent across different contexts and populations.

Second, the principal investigator (AA), who conducted all interviews, works within the same institution as the participants. This positionality may have influenced participants’ willingness to disclose experiences due to perceived power differentials or social desirability. To mitigate bias, AA emphasized confidentiality and voluntary participation. To enhance reflexivity and analytic rigor, the data collection and analysis processes were reviewed and supervised by experienced researchers external to the institution; however, the potential for social desirability bias during the interviews cannot be ignored. Moreover, given the hierarchical nature of clinical training and regional cultural norms, participant responses may have been influenced by perceived power differentials. These contextual features should be considered when interpreting the transferability of our findings.

Third, we acknowledge the potential for researcher bias arising from the involvement of a single researcher (AA) in conducting the interviews, transcribing the data, and performing the initial coding. While these overlapping roles may have influenced data collection and interpretation, analytic decisions were reviewed collaboratively with the research team to enhance reflexivity, transparency, and methodological rigor. Nonetheless, the influence of the researcher’s perspectives and experience on the findings cannot be fully excluded.

Forth, the data were based solely on interview narratives, and no direct observational data were used to triangulate the findings. This may affect the robustness of interpretations, especially regarding tacit or unspoken elements of supervisor–trainee interaction. Future research employing ethnographic or mixed-method approaches could yield a more nuanced understanding of the trust-building process in context.

Finally, the discontinuity of the supervisor–trainee relationship in this setting, often limited by short rotations and systemic disruptions, may differ from contexts with more longitudinal training models. This may limit the external validity of the findings, especially in systems where continuity and team-based care are more common. Further research should explore how duration and quality of the supervisor–trainee relationship influence trust formation across different national and institutional contexts.

## Conclusion

This study reveals that novice supervisors in family medicine construct trust as a dynamic, relational, and context-sensitive process, shaped by evolving impressions, observed behaviors, and perceived clinical risk. Trust decisions were not solely based on trainees’ competence, but emerged through a complex interplay of supervisor characteristics, task demands, trainee behaviors, and institutional constraints. Novice supervisors often began from a position of caution, adopting a “more supervision/less trust” approach, especially in high-risk or ambiguous settings. Over time, familiarity and repeated observation facilitated progressive entrustment, although this process was frequently hindered by contextual factors such as short rotation lengths, high workload, and legal or logistical limitations.

The findings underscore the need for tailored faculty development programs that support novice supervisors in developing assessment literacy, trust calibration skills, and strategies for navigating dual roles. Systems-level interventions (i.e., longitudinal supervision models, manageable supervisor–trainee ratios, and structured training model) are essential to promote safe, learner-cantered autonomy. Ultimately, strengthening supervisors’ ability to make defensible trust decisions can enhance both educational quality and patient care outcomes.

## Data Availability

The raw data supporting the conclusions of this article will be made available by the authors, without undue reservation.
