# Supplementary material for: Factors influencing novice supervisors’ trust decisions in postgraduate family medicine training: a qualitative study
Source: Front Med (Lausanne). 2026 Feb 4;13:1712735. doi: 10.3389/fmed.2026.1712735 (PMC12913528; doi:10.3389/fmed.2026.1712735)
Supplement: Supplementary file 1 [file Table_1.docx]

**Appendix 1: Interview Guide**

Thank you for participating in this interview. We appreciate your time and willingness to share your thoughts and experiences. Our goal is to understand, from your perspective as a supervisor of postgraduate residents of family medicine, how you interact with, supervise and trust a resident. The purpose of this study is to understand the process of supervision, not to gather any information about specific residents. Please do not use any team members or patient names or identifying information. I would like to record this interview and have it transcribed. Your name and any identifying information will be removed from the transcript and will not be used during the analysis.

**---Permission to record the interview---**

We know that supervisors are making decisions about when to trust their resident to act independently and when to supervise more closely. We are interested in learning about supervisors’ decisions about when to give residents more or less independence. Please focus your answers on your experience with your current residents (not the interns).

**Interview Questions:**

1. What does it mean to you to trust a resident with patient care responsibilities?
2. How do you decide when you are comfortable letting the resident work independently?
3. What makes you decide if you trust a resident with a certain task or not?
4. Can you give me an example of a time when you decided to trust a resident with a certain task to be performed independently?
5. How about a specific time you decided not to trust a resident with a certain task?
6. With what tasks or aspects of patient care do you feel comfortable that you can rely on the resident to complete independently?
7. Are there situations where you have not been certain how much supervision you needed to provide? Can you tell me about that?
8. I’d like to ask a bit about your working relationship with your residents. Do you think your relationship with the residents affects your trust in their abilities? (in positive or negative way)

- If yes, then why?
- If not, then why not?

1. Are there any other factors related to your relationship with residents that help you trust them?
2. There can be particular resident characteristics that influence how much you feel you need to supervise. Are there any resident traits or behaviors that affect your style or level of supervision?
3. Talking about the residents you recently worked with, were there times that you felt your resident should have sought help but did not? Can you tell me about that?
4. We recognize that issues related to the particular situation can affect the way that you interact with your residents. What particular situational issues influence your trust on your resident working independently?
5. How did those issues influence your level of supervision?
6. When do you feel that you have had enough experience with a resident to determine whether or not you can trust him/her to complete tasks independently?
7. Is there anything else you’d like to add about what it means to you to trust a resident with patient care responsibilities?
